# Carbon Fluxes between Primary Metabolism and Phenolic Pathway in Plant Tissues under Stress

**DOI:** 10.3390/ijms161125967

**Published:** 2015-11-04

**Authors:** Sofia Caretto, Vito Linsalata, Giovanni Colella, Giovanni Mita, Vincenzo Lattanzio

**Affiliations:** 1 Institute of Sciences of Food Production, National Research Council, Via Provinciale Lecce-Monteroni, 73100 Lecce, Italy; sofia.caretto@ispa.cnr.it (S.C.); gianni.colella@ispa.cnr.it (G.C.); giovanni.mita@ispa.cnr.it (G.M.); 2 Institute of Sciences of Food Production, National Research Council, Via Amendola, 122/O, 70126 Bari, Italy; vito.linsalata@ispa.cnr.it; 3 Department of Sciences of Agriculture, Food and Environment, University of Foggia, Via Napoli 25, 71100 Foggia, Italy

**Keywords:** environmental stresses, phenolics, resistance costs, trade-offs, proline, transduction pathway

## Abstract

Higher plants synthesize an amazing diversity of phenolic secondary metabolites. Phenolics are defined secondary metabolites or natural products because, originally, they were considered not essential for plant growth and development. Plant phenolics, like other natural compounds, provide the plant with specific adaptations to changing environmental conditions and, therefore, they are essential for plant defense mechanisms. Plant defensive traits are costly for plants due to the energy drain from growth toward defensive metabolite production. Being limited with environmental resources, plants have to decide how allocate these resources to various competing functions. This decision brings about trade-offs, *i.e.*, promoting some functions by neglecting others as an inverse relationship. Many studies have been carried out in order to link an evaluation of plant performance (in terms of growth rate) with levels of defense-related metabolites. Available results suggest that environmental stresses and stress-induced phenolics could be linked by a transduction pathway that involves: (i) the proline redox cycle; (ii) the stimulated oxidative pentose phosphate pathway; and, in turn, (iii) the reduced growth of plant tissues.

## 1. Plant Phenolic Secondary Metabolites

Higher plants produce a bewildering number of chemical compounds (more than 200,000 different structures). These compounds can be classified as belonging to primary or secondary metabolites, also called natural products. Primary metabolites are ubiquitous in plants and fulfill essential metabolic roles. Natural products refer to compounds that are differentially distributed in the plant kingdom and fulfill a very broad range of physiological roles that are considered essential for their adaptive significance in protection against environmental constraints. Nowadays, it is widely recognized that natural products play a role in plant growth, reproduction, and the continued survival of land plants [1,2,3].

Plants exhibit a variable qualitative and quantitative distribution of natural products in different tissues and organs. This variability is also observed between different physiological stages, between individuals, and between populations [4,5,6,7,8]. Plants synthesize amounts of natural products under genetic control upon environmental stimuli. These natural products are synthesized in plants through metabolic pathways, which are an integral part of the whole plant developmental program, as a response to stress conditions induced by biotic and abiotic agents. A strict genetic and epigenetic control of these pathways guarantees the proper production profile of different secondary metabolites. Their transport represents an additional level of regulation [9,10,11,12,13,14,15].

**Figure 1:**
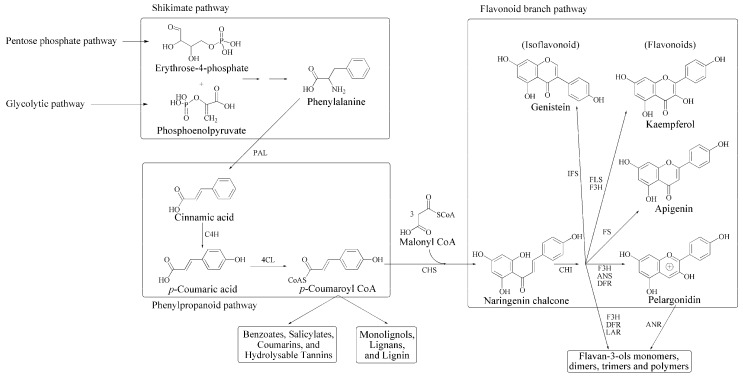
General biosynthetic pathway of phenylpropanoid and flavonoid structures. PAL, phenylalanine ammonia-lyase; C4H, cinnamate-4-hydroxylase; 4CL, 4-coumaroyl:CoA-ligase; CHS, chalcone synthase; CHI, chalcone isomerase; ANS, anthocyanidin synthase; DFR, dihydroflavonol reductase; FS, flavone synthase; FLS, flavonol synthase; F3H, flavanone 3-hydroxylase; IFS, isoflavone synthase; ANR, anthocyanidin reductase; LAR, leucoanthocyanidin reductase (redrawn from [26] with permission of Elsevier).

Plant phenolics are the most widely distributed natural products. In leaf extracts of vascular plants several classes of phenolic compounds such as esters, amides, and glycosides of hydroxycinnamic acids, flavonoids, proanthocyanidins, and their relatives can be found. In addition, polymeric phenolics, such as lignin, suberin, and melanins, can be commonly found in these plants [16,17,18,19]. (Poly)phenolic compounds are produced in plants by the sequential action of five biosynthetic pathways. The glycolytic and pentose phosphate pathways provide precursors (phosphoenolpyruvate and erythrose-4-phosphate, respectively) to the shikimate pathway. Phenylalanine, produced by the shikimate route, is the precursor of phenylpropanoid metabolism which, in turn, feeds the diverse specific flavonoid pathways (Figure 1) [20,21,22,23,24,25,26].

Phenolic compounds have been produced in plants because of the interactions with the challenging environment throughout the course of evolution. This production has been an event of paramount importance for the colonization of land. In this connection, plant phenolics represent a noticeable example of plant metabolic plasticity that enable plants to survive environmental stresses. Indeed, when the first plants moved from water to land, they were forced to cope with stressful conditions, such as ultraviolet (UV) radiation [8,27,28,29]. At that time, the ability of UV radiation to severely damage biomolecules induced land plants to synthesize phenolic molecules (starting from the shikimate pathway, Figure 1) to be used as sunscreens, about 480–360 million years ago. In algae, the shikimate pathway only produces phenylalanine and tyrosine, which are already present in proteins of primordial bacteria. Aerobic bacteria and algae produce polyketides through the condensation of acetyl-CoA as a starter unit and malonyl-CoA for chain elongation. In bryophites, the starter unit acetyl-CoA was substituted by cinnamoyl-CoA, leading to flavones and flavonols, which, absorbing UV light, act as photoscreens in all terrestrial plants [30,31,32].

Besides UV radiation, other stress factors are present in an aerial environment which require the adaptation of plant metabolism. Once more, polyphenol chemistry is involved in the adaptation to these environmental stressors. For example, the polymerization of catechins, resulting from leucoanthocyanidins/anthocyanidins submitted to reductive reactions, produces condensed tannins, which have an important role in defending plants against viruses, bacteria, fungi, insects, and herbivores [26,33,34].

## 2. Plant Phenolics and Their Role in Defense against Environmental Stresses

Environmental constraints such as drought, heat, salinity, cold, high light/UV-B, heavy metals, air pollution, nutritional deficiency, insect pests, and pathogens result in a harmful impact on plant growth and yield under field conditions [35,36,37]. Therefore, to cope with these conditions, plants must promptly identify environmental stresses and then activate defense responses. Environmental stress in plants induces changes in growth conditions, altering or disrupting their metabolic homeostasis. In these conditions, plant metabolism must be modified to make it possible to produce compounds necessary to cope with the stress. Such an adjustment of the metabolic pathways is usually referred to as acclimation. Changes of cellular and molecular activities represent plant strategies of adaptation to stress [38,39].

Higher plants accumulate a very large number of different (poly)phenolic structures that are believed to act as defense compounds against abiotic and biotic stresses [35,40]. Both constitutive and induced defenses are involved in the optimal protection of a plant against environmental stressors [41]. To understand and improve plants’ stress responses and tolerances, researchers have focused on the signaling perception, transcriptional regulation, and expression of functional proteins in the stress response mechanisms. The accumulation of small molecules with antioxidative activity has often been discussed with respect to their role in mitigating the accumulation of reactive oxygen species (ROS) induced by stresses.

In the natural environment, plants come across several pests and pathogens. Plant defense toward potential pathogens includes both the rapid strengthening of pre-existing physical and chemical barriers and/or the *de novo* synthesis of a large number of defensive compounds through the induction of gene expression. The successful defense of a plant results in the restriction of fungal growth, which is usually caused by different defensive responses, such as the production of the so-called “phytoalexins” and pathogen-related proteins, and the accumulation of phenolic substances in the cell wall [42,43,44,45,46]. As far as pest attack is concerned, it should be stressed that the chemical composition of plant tissues is the main factor, together with physical factors, that influences insect acceptance or rejection of the plant as food. Phenolics also prevent insect oviposition on the host plant, as well as larval growth. It is well studied, for example, that flavonoids negatively influence the growth and development of various insects [47,48,49]. Tannins also may have a negative effect on insect growth due to their astringent taste, their ability to produce complex proteins, thus reducing digestibility, and their ability to act as enzyme inactivators. Recent papers [50,51,52,53,54,55] dealing with tannin oxidation in insects suggest that these oxidation reactions should also be considered as a plant defense mechanism [15]. The production of chemical defenses is expensive for plants due to the energy needed for their biosynthesis. To save these costs plants can produce chemical defenses just after an initial attack by a pathogen or insect. However, this strategy may not be effective if the attack is rapid and severe. Thus, plants exposed to frequent attacks invest resources in constitutive defenses, while plants that are subjected to rare attacks can rely on induced defenses [41,56,57].

Light is a fundamental important environmental signal regulating plant development and gene expression [58]. Elevated UV-B radiation that can be a consequence of ozone depletion has pleiotropic effects on plant life [59,60]. The most common effects are plant growth reductions and increased quantities of phenolic compounds in plant tissues [61]. Indeed, as for many abiotic stresses, ROS production is involved in UV radiation stress. A general strategy adopted by plants is to scavenge ROS using both enzymatic and nonenzymatic scavengers such as phenolic compounds [62,63]. Phenolic accumulation is induced by UV-B activation of the phenolic biosynthetic pathway. The presence of increased levels of phenolics in the epidermal cells results in hampering UV-B penetration, thus protecting the photosynthetically active tissues [64].

Both the primary and secondary metabolism of higher plants are influenced by mineral nutrition. In most terrestrial ecosystems, plant growth is nitrogen (N)-limited, but phosphorus (P)-limitation also occurs frequently. In these conditions, high concentrations of phenolics in tissues of low-productivity species growing at infertile sites are observed [65,66]. Broadly, it was observed that low-productivity species have higher amounts of secondary compounds than high-productivity species. Species growing in nutrient-poor habitats often have traits that lead to high nutrient retention and high levels of secondary metabolites, which have a defense role against herbivores and pathogens [67,68,69,70]. Deficiencies of essential elements (such as N, P and potassium (K)) can increase the amounts of phenolics in plant tissues either as existing pools or by inducing their *de novo* synthesis [71,72,73]. Barley plants grown under nitrogen deficiency conditions showed lower biomass, while leaf levels of soluble phenolics increased [74]. Iron deficiency induced increased amounts of phenolic acids in root exudates of non-graminaceous monocots and dicots [75,76,77]. An increased amount of anthocyanins is recognized as a consequence of P limitation. Anthocyanin over-accumulation lowers the accumulation of ROS *in vivo* under oxidative and drought stress [78]. As a consequence of P limitation, the content of phenylpropanoids and flavonoids resulted in increased *Arabidopsis thaliana* roots and shoots. The overexpression of MYB transcription factors PAP1/MYB75 and/or PAP2/MYB90 led plants to increase the content of anthocyanins and glycosides of quercetin and kaempferol [79,80]. This indicates that PAP1 and PAP2 have a role in increasing phenolics during P limitation [70,81,82].

Different hypotheses, such as the carbon-nutrient balance hypothesis and the growth-differentiation balance hypothesis, have been considered in order to explain the influence of nutrient deficiency on secondary phenolic metabolism. These hypotheses affirm that carbon skeletons synthesized by photosynthesis are dynamically used for growth (primary metabolism) or defense (secondary metabolism). Because allocation for plant growth and defense can take place at the same time in plants, these hypotheses suggest that secondary metabolism utilizes extra carbon skeletons when growth is more limited than photosynthesis (e.g., due to mineral element deficiencies) [57,67,83,84,85].

Low temperatures are another important plant abiotic stress. Lower temperatures alter the membrane structure, also affecting the activity of membrane-bound enzymes. An excessive production of ROS can be associated with chilling and this has deleterious effects on membranes. Moreover, low temperatures reduce scavenging enzyme activities, impairing the whole antioxidant plant response. In these conditions, some plants can adapt by modifying the membrane composition and activating oxygen-scavenging systems [86,87,88]. Low temperature conditions also determine the increased production of phenolics, which exert antioxidant activity in chilled tissues. An enhancement of phenylpropanoid metabolism is induced in plant tissues when temperatures decrease below a certain threshold value [15,89,90]. Low, non-freezing temperature stress induces an increase in phenylalanine ammonia-lyase and chalcone synthase activities, as well as the activation of a number of genes involved in phenolic metabolism [91]. Anthocyanins are believed to accumulate in leaves and stems of *Arabidopsis thaliana* in response to low temperatures [92,93,94]. Christie *et al.* [95] show that an increase in anthocyanin and mRNA abundance in the sheaths of maize seedlings are positively related with the severity and duration of the cold. 

Finally, transition metals also cause oxidative stress in plants. Once again, transition metals most likely promote the formation of hydroxyl radical production. Available data suggest that heavy metals such as copper and cadmium, if they are not detoxified soon enough, may activate various reactions that, by disrupting cell redox control, lead to the inhibition of plant growth, the stimulation of secondary metabolism, and lignin deposition [96].

## 3. Costs of Resistance

Plant growth and development are dependent on the availability of essential environmental resources such as light, water, and nutrients. Usually, plants have to find a balance in the allocation of these resources to various physiological functions, such as growth and defense. Allocation theory in plant physiology assumes that plants have a limited supply of essential resources, which they must split between different competing physiological functions, such as growth, maintenance, and reproduction. These functions are mutually exclusive, since allocation to one function directly results in a decrease in the allocation to other functions, and consequently, an optimal pattern of allocation will exist. This multiple use of limited resources creates resource allocation trade-offs. It has been speculated that the process of allocation and trade-offs between various activities and functions must have been improved by natural selection. Because of these trade-offs between a plant’s various functions, the concept of costs and benefits helps explain allocation patterns at both the physiological and evolutionary level [97]. External resources from the environment are devoted to internal needs, including growth, survival, and reproduction, as well as to physiological and genetic mechanisms of acclimation and genetic adaptation to the environment. Such a balance is continuously threatened by the occurrence of abiotic and biotic stress conditions. Hence, plants have also to devote a number of their resources to stress defense [15,98,99,100,101].

Plant defensive traits are costly for plants because of the energy needed for the biosynthesis of defensive compounds [84,102,103,104]. Hence, plants could struggle with the choice of allocating resources to different competing needs, creating possible trade-offs, *i.e.*, promoting some functions and neglecting others as an inverse relationship. Much research has been carried out for quantifying these costs in plants, *i.e.*, to link an evaluation of plant performance (in terms of growth rate) with levels of defense-related metabolites. Zangerl *et al.* [105] investigated the effects of the stress-induced defensive furanocoumarins on plant growth over a four-week period in wild parsnip. They found that total biomass and root biomass were reduced by 8.6% and 14%, respectively, in plants that had 2% of their leaf area removed compared to intact plants. At the same time, they also found an increase in furanocoumarin production. Pavia *et al.* [106] investigated the balance between phlorotannin production and plant growth by measuring phlorotannin content and annual growth in *Ascophyllum nodosum*. These authors found a significant negative correlation between phlorotannin content and plant growth. In good agreement with these data, allocation theory expects a trade-off mechanism between plant growth and defense needs, which allocates carbon between the primary and secondary metabolism, and that, in turn, provides the plant with an adequate adaptation mechanism against environmental stresses [56,84,97,98,107,108,109,110,111,112,113,114].

Primary metabolism provides carbon skeletons for the biosynthesis of phenolic metabolites (Figure 1), which are involved in several functions in signaling and defense against abiotic and biotic stress. Primary metabolism needs large amounts of the available plant resources. Therefore, when the growth rate is high, the production of phenolic compounds could be impaired by the shortage of substrates [115]. Plants producing defense compounds need to devote their limited resources to survival functions, being forced to make the choice between growing and defending, thus diverting carbon skeletons from the primary to secondary metabolism [84,98]. Therefore, plant metabolism must possess adequate flexibility to adapt to changes during development and to face environmental challenges. To this purpose, several mechanisms can be involved, including the alteration of enzyme kinetics as a reaction to metabolite level and/or induced gene transcription [116,117,118,119,120,121].

The “growth *vs.* defense” allocation dilemma has gained great interest in plant ecophysiology, even if specific plant choices that are the result of adaptation to particular environmental conditions are not definitely comprised [84,122,123,124,125,126]. The plant responses to environmental stress include biochemical and molecular mechanisms by which plants recognize and transfer the signals to cellular machinery, thus triggering adaptive reactions. Investigating mechanisms of stress signal transduction is greatly important in developing strategies for improving crop stress tolerance [15,127,128,129]. 

An increased level of phenolic metabolites in plant tissues is a peculiar trait of plant stress. Quantitative (pre-existing phenolics) and qualitative (induced phenolics, *de novo* synthesis) changes in phenolic composition confer to plants’ various physiological functions that are useful for adapting to environmental disturbances [42,70,92,93,116,130,131,132,133,134,135,136,137,138,139,140]. Indeed, it must be stressed that the enhanced production comes from the enhanced activity of the enzymes involved in the phenolic pathway, including phenylalanine ammonia lyase and chalcone synthase. In addition, the enzyme activity of PEP (phosphoenolpyruvate)-carboxylase is enhanced, and this suggests a reallocation from sucrose production to defensive metabolite production [15,98,101,141,142,143,144,145,146,147,148].

**Figure 2:**
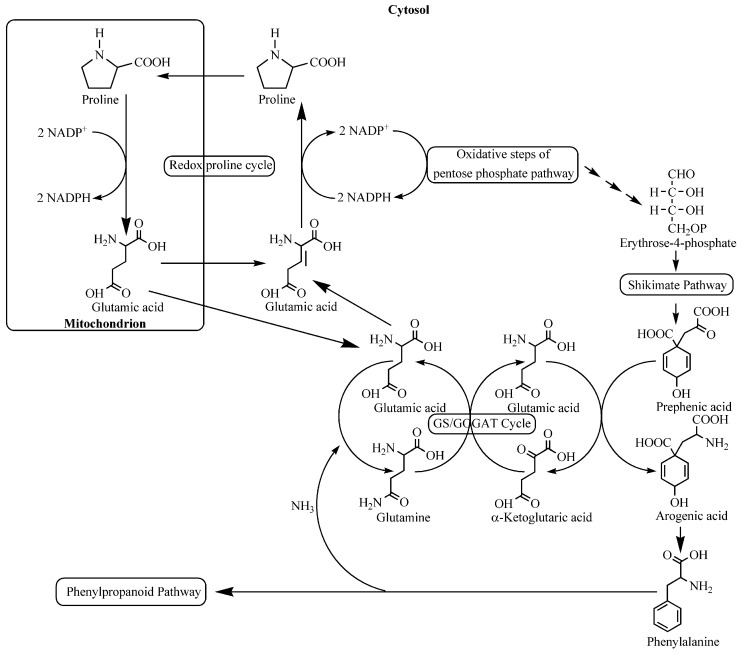
Relationships between primary and secondary metabolism and role of endogenous proline in stimulating phenylpropanoid pathway. GS, glutamine synthetase; GOGAT, glutamate synthase (redrawn from [115] with permission of Elsevier).

What about the link between environmental stress and adaptive responses of plants to stress? Lattanzio *et al.* [115] suggest a scheme (Figure 2), which combines the amino acid proline, which is known to be induced by stress, with energy transfer to phenylpropanoid biosynthesis via the oxidative pentose phosphate pathway (OPPP) [149]. In plant tissues, an accumulation of free proline can be induced by many biotic and abiotic stresses. In this regard, it has also been suggested that the level of proline induced by stress conditions could be mainly mediated through the influence of its synthesis and degradation on cellular metabolism [115,150,151,152,153]. Most published papers have supported the role of proline as a mediator of osmotic imbalance, a free radical scavenger, and a source of reducing power. Proline also protects enzymes and membranes during changes of environmental conditions [154,155,156]. Proline action is also typical of a signaling molecule modulating cell physiology by inducing the expression of specific genes necessary for the plant stress response [157]. Moreover, it must be emphasized that stressed plants are often subjected to an excessive exposure to light, more than is needed for photosynthesis. When this occurs, the reduced regeneration of NADP^+^ during photosynthetic carbon fixation results in cellular redox imbalance. Some studies suggest that a stress-induced increase in the transfer of reducing equivalents into the proline synthesis and degradation cycle should permit sensitive regulation of cellular redox potential in cytosol by enhancing the NADP^+^/NADPH ratio. The increased NADP^+^/NADPH ratio possibly enhances the OPPP activity, providing precursors required for the increased demand of phenolic metabolites to be produced during stress [151,155]. The alternating oxidation of NADPH by proline synthesis and the reduction of NADP^+^ by the two oxidative steps of the OPPP serve to link both pathways, and this allows the maintenance of the high speed of proline production during stress.

## 4. Nutritional Stress Induces Supply Pathways from Primary Metabolism to Phenolic Secondary Product Formation

In plant tissues, increased amounts of phenolics observed under environmental stress can be considered both a common response of plant adaptation to stressful conditions, improving evolutionary fitness, and a way of channeling and storing carbon skeletons produced by photosynthesis during periods when plant growth is curtailed. The induction gene expression of phenolic metabolite pathways by biotic and abiotic stress is often acted by signaling molecules such as salicylic acid and jasmonic acid [3,14,15,158,159,160].

Both the OPPP and Calvin cycle can provide carbon skeletons in the form of erythrose-4-phosphate, which, together with glycolysis-derived phosphoenolpyruvate, acts as a precursor for phenylpropanoid metabolism via the shikimic acid pathway (Figure 1). In addition, it has been suggested that OPPP provides reducing equivalents to be used for the biosynthesis of phenolic compounds. Furthermore, OPPP activity results are enhanced when carbon flux into the phenylpropanoid pathway is also enhanced [115,161,162,163].

Lattanzio *et al.* [115] studied the influence of stress-induced synthesis of defensive phenolics on the growth of oregano (*Origanum vulgare* L.) shoots grown on Murashige and Skoog medium (MS) or half-strength MS medium. The growth rate and total phenolic content were shown to be significantly negatively correlated. Nutritional deficiency decreased the fresh biomass of oregano shoots (−40%) in comparison with the control (MS). On the contrary, nutritional stress induced a significant increase of both the total phenolic content (+120%) and rosmarinic acid, the most representative phenolic compound in oregano shoot extracts (+158%). The intracellular free proline content was also found increased (+31%). It should be noted that this moderate increase of endogenous proline in stressed tissues could be related to its consumption in increasing the net flux through the proline cycle (see Figure 2). Figure 2 also suggests a link between elicited proline and increased phenolic metabolism via the replacement of the NADP^+^ delivery to OPPP which, successively, provides NADPH and carbon skeletons in the phenylpropanoid pathway [162,164,165,166].

**Figure 3:**
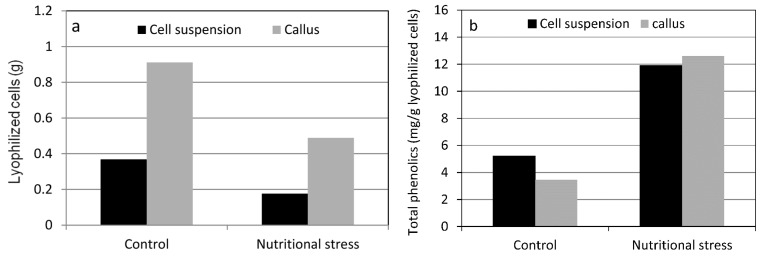
Response of artichoke cells to nutrient deficiency. Cell growth (**a**) and total phenolic content (**b**). Unpublished data.

Similar results have been observed when callus and cell suspension cultures of artichoke (*Cynara cardunculus* L. subsp. *scolymus* (L.) Hayek) subcultivated in Gamborg B5 medium (control) or in half-strength Gamborg B5 medium (nutritional stress) have been used as a model system. Both callus and cell suspension cultures suffered relevant changes when subjected to nutritional stress: they accumulated secondary metabolites and, meanwhile, their growth was negatively affected by stress conditions. Figure 3 shows the existence of a negative correlation between the growth rate (Figure 3a) and total phenolic content (Figure 3b) in cell cultures of artichoke. The growth rate of stressed cell suspension cultures was reduced by 52% compared to the non-stressed control. In contrast, the total phenolic content was enhanced by nutrient deficiency by 2.3-fold compared to the control level after a 30-day treatment. The same results were observed with artichoke callus cultures. Following nutrient deficiency, the growth of callus cultures was reduced by 47% compared to the control and this reduction seemed to be related to an energetic drain involved in generating the increased level of phenolic metabolites (3.6-fold greater than the control level) which diverts resources from the biomass production. This evidence confirms the theoretical predictions that a trade-off exists between growth rate and defensive secondary metabolite investment when plant cells are in low-resource habitats [78,167]. Again, in order to understand the biochemical levels of regulatory mechanisms that control carbon fluxes between the primary and secondary metabolism, the role of proline, which figures prominently in most stress-mediated responses [115,168,169], has been also considered. The analysis of free proline content in cultivated artichoke cells shows an increase in proline level (by 38%–50% compared to the control) in response to nutritional stress. As already hypothesized for stressed oregano shoots, once again, it can be assumed that the observed increase of proline in artichoke cells subjected to nutrient deficiency could be explained by its rapid utilization in the proline redox cycle (see Figure 2).

Broadly, in case of limited resources, plants need a well-balanced trade-off which permits growth without excluding defense responses. Various hypotheses have been proposed in order to elucidate the influence of environmental constraints on the trade-off between growth and defensive compounds. Some authors propose that it is the possibility of a trade-off between growth and differentiation (*i.e.*, biosynthesis of natural products, including phenolics) [67,84,170,171]. An alternative model [172,173,174] suggests a competition between protein and phenylpropanoid synthesis for using of the precursor phenylalanine. Therefore, at a high growth rate the synthesis of proteins reduces the availability of phenylalanine or phenolic biosynthesis. However, this model does not explain whether the protein synthesis and the synthesis of phenolics use the same pool of phenylalanine, or two separate pools.

Nowadays, the theory that growth and defense interact within the plant and compete for limited resources is considered a well-established principle. Because there are trade-offs between a plant’s various functions, the concept of costs and benefits helps explain allocation patterns at both the physiological and evolutionary levels. One trade-off implies that constitutive or induced defenses need resources that could otherwise be devoted to growth and development. Comparisons among species suggest that high levels of defensive compounds are associated with resource-limited environments. Species adapted to low-nutrient availability generally have higher defense allocations than species of resource-rich habitats. In conditions of nutrient deficiency, if plants maintain defensive compound levels as nutrient resources decline, then growth and other competing physiological processes may decrease [65,67,97,110,144,175,176,177,178]. Here, it must be stressed that the phenolic metabolism is not only a feature of normal development but can also be induced by environmental stress conditions. Any new knowledge concerning responses of plant cell systems to real-life obstacles will help to improve our understanding of how plants work and how their resistance and/or tolerance to environmental stresses can be improved. In addition, this new knowledge can help to understand biochemical and molecular levels of regulatory mechanisms [179].

The above data are consistent with a biochemical regulatory mechanism proposed by Lattanzio *et al.* [115] (Figure 2). After the application of a nutritional stress, the growth rate of cell and tissue cultures is reduced and this effect can be related to an energetic drain that redirects resources from biomass production. At the same time, the imposed nutritional stress induces an increase of intracellular proline, which improves the tolerance to ROS produced by stressed cell and tissue cultures [180,181]. 

It should also highlight that the increased synthesis of proline maintains NAD(P)^+^/NAD(P)H ratios at values compatible with cell metabolism under normal conditions. This adjustment could be considered a metabolic response which elicits the signal transduction pathway between the perception of nutritional stress and the adaptive physiological response. In addition, the increased NADP^+^/NADPH ratio, caused by proline synthesis, increases the activity of the OPPP. Mitochondrial proline oxidation can also affect the OPPP by recycling glutamic acid into the cytosol to generate a proline redox cycle [151]. Finally, glutamic acid could also be used to recycle ammonium ions, generated in the reductive deamination of phenylalanine, by means of the glutamine synthetase and glutamate synthase (GS/GOGAT) cycle [182,183].

## 5. Concluding Remarks

The resources to be managed by plants are carbon, nutrient elements, water, and energy. Management here means the allocation of resources to fundamental functions and to acclimate to the environment. Noticeably, such forms of allocation imply that the plant must make decisions. These decisions depend both on the plant’s current developmental and metabolic status and on the environmental circumstances. For survival, plants need to regulate various requirements by means of resource allocation, estimating different sources and drops in resource fluxes *versus* the constraints associated with them [101].

When resources are limited, plants with naturally slow growth are favored over those with fast growth rates; slow growth rates, in turn, promote large investments in defense compounds [98,184]. Plant phenolics are defensive compounds that often accumulate in vegetative tissues when plants are subjected to different types of stress conditions. Whether and how stress-induced phenolics divert carbon skeletons from the primary metabolism and act as stress-protective molecules have been a subject of debate. As previously stated, phenolic levels increase during stress since growth is inhibited more than photosynthesis. Therefore, the photosynthates produced are redirected to the secondary metabolism [81]. Alternatively (or in addition), it could be suggested [15] that a peculiar feature of plant metabolism is the flexibility that allows it to respond to the environmental changes through developmental changes: adaptation strategies to environmental stress are costly and this could result in growth limitations.

Results discussed in this review support the hypothesis that there is a trade-off between growth and defense in plant cells (tissue and cell cultures) and that the trade-off is mediated by resource availability. Data also suggest that nutritional stress and stress-induced phenolics are linked by a transduction pathway that involves: (i) the proline redox cycle; (ii) the stimulated oxidative pentose phosphate pathway; and, in turn, (iii) the reduced growth of callus and cell suspension cultures.
